# Shining in the dark: the big world of small peptides in plants

**DOI:** 10.1007/s42994-023-00100-0

**Published:** 2023-04-08

**Authors:** Yan-Zhao Feng, Qing-Feng Zhu, Jiao Xue, Pei Chen, Yang Yu

**Affiliations:** Guangdong Key Laboratory of Crop Germplasm Resources Preservation and Utilization, Key Laboratory of South China Modern Biological Seed Industry, Ministry of Agriculture and Rural Affairs, Agro-Biological Gene Research Center, Guangdong Academy of Agricultural Sciences, Guangzhou, 510640 China

**Keywords:** Small peptide, Biological function, Regulatory mechanism, Crop improvement

## Abstract

Small peptides represent a subset of dark matter in plant proteomes. Through differential expression patterns and modes of action, small peptides act as important regulators of plant growth and development. Over the past 20 years, many small peptides have been identified due to technical advances in genome sequencing, bioinformatics, and chemical biology. In this article, we summarize the classification of plant small peptides and experimental strategies used to identify them as well as their potential use in agronomic breeding. We review the biological functions and molecular mechanisms of small peptides in plants, discuss current problems in small peptide research and highlight future research directions in this field. Our review provides crucial insight into small peptides in plants and will contribute to a better understanding of their potential roles in biotechnology and agriculture.

## Introduction

Small peptides are a novel class of small proteins in organisms that are translated directly from small open reading frames (sORFs) in a noncoding region or generated indirectly via processing of precursor proteins. Small peptides are not easy to identify because gene annotation and protein analysis programs are often optimized for identifying large proteins. Moreover, as some sORFs do not begin with an AUG start codon, they are difficult to distinguish from noncoding RNAs or small peptide-encoding ORFs. Overall, most sORFs are functionally uncharacterized so that small peptides are regarded as “dark matter” in plants, and their potential has been underestimated for some time.

Due to rapid advancement of next-generation sequencing (NGS), mass spectrometry (MS) and novel algorithmic tools, many small peptides have been discovered in *Arabidopsis thaliana*, *Oryza sativa* (*O. sativa*) (Chen et al. 2020a; Fang et al. 2019; Jin et al. 2022; Wang et al. 2020a), *Zea mays* (*Z. mays*) (Frank and Smith 2002; Liang et al. 2021; Wang et al. 2009a, 2020b), *Mus musculus* (*M. musculus*) (Anderson et al. 2015; Bi et al. 2017) and *Vitis vinifera* (V*. vinifera*)(Chen et al. 2020b), suggesting a hidden layer of precise regulation networks in life processes. Recent reports have provided evidence showing that small peptides play important roles in physiological activities such as abiotic stresses, plant growth, organ morphogenesis, and sexual reproduction.

Current criteria for the definition of small peptides are not unified. Most regard proteins with 100 aa or less as small peptides (Hellens et al. 2016; Hsu and Benfey 2018; Ong et al. 2022), whereas some studies on small peptides involve those more than 100 aa long (Hu et al. 2021; Matsubayashi 2014). For a comprehensive summary of the landscape of small peptides in plants, we in this paper consider proteins with no more than 150 aa as small peptides. Owing to their small size, the methods for characterizing small peptides and their functions are somewhat different from those used to study general protein-coding genes. Therefore, systematic reviews are needed to provide insight into plant small peptides, promoting intensive study in this field. Herein, we discuss the classification and progress made in studying the molecular and biological functions of plant small peptides. Furthermore, we summarize strategies for studying small peptides in plants. Finally, we discuss extended applications of plant small peptides. We believe that this review will facilitate research on this novel class of plant regulators.

## Classification and biosynthesis of small peptides

Small peptides can be generated from various regions within plant genomes. In addition to protein-coding genes, noncoding RNAs that were once considered incapable of being translated have also been found in recent years to encode small peptides. Small peptides are translated in the cytosol and then transported to the locations where they function. Therefore, they can be classified into protein-coding/noncoding gene-derived small peptides according to their genomic origin or can be designated secretory/nonsecretary peptides according to their distribution.

### Small peptides derived from noncoding regions

**Fig. 1:**
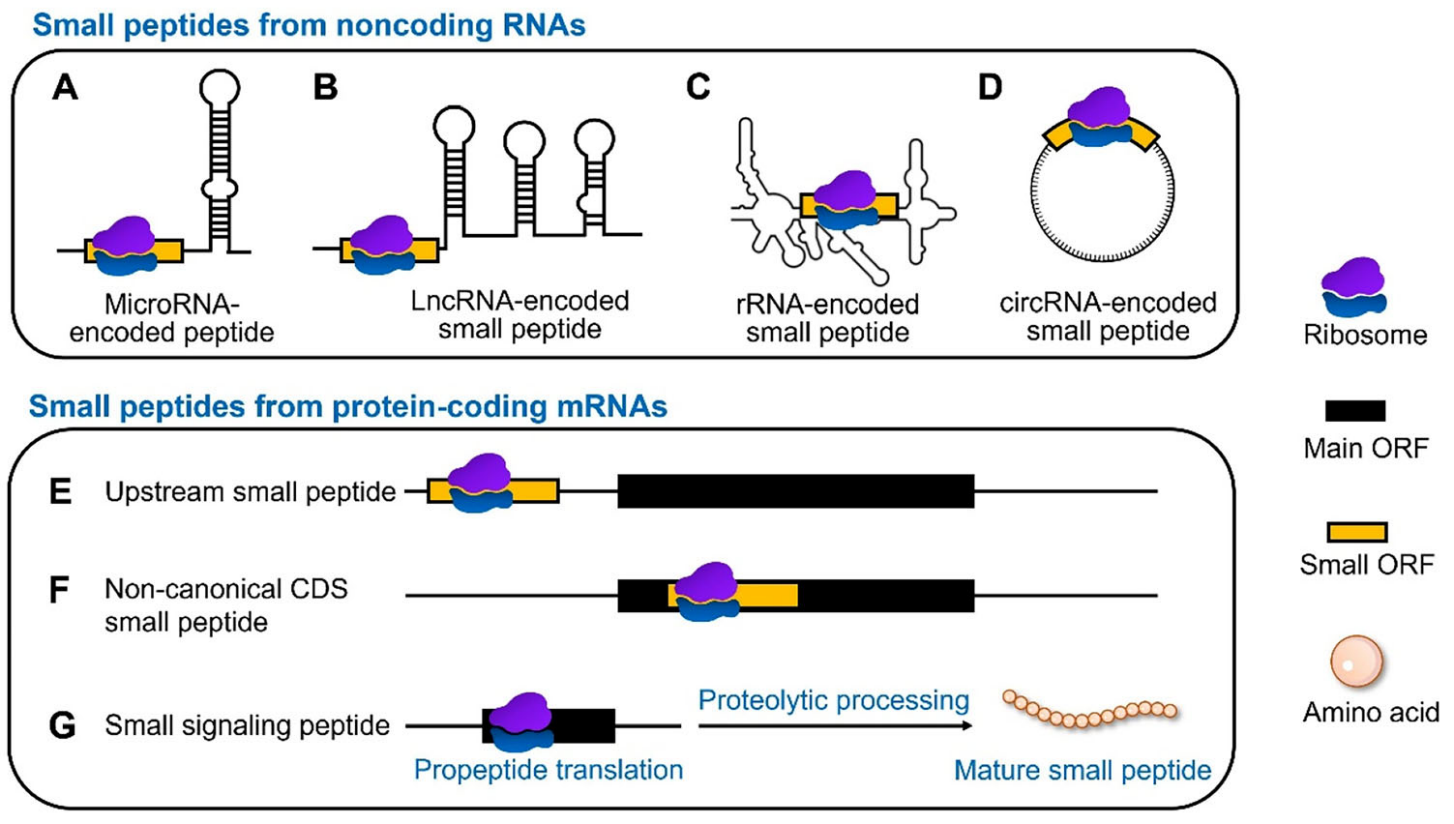
Schematic of small peptides from different genomic origins. **A** MicroRNA-encoded peptide. **B** LncRNA-encoded small peptide. **C** rRNA-encoded small peptide. **D** CircRNA-encoded small peptide. **E** Upstream small peptide. **F** Noncanonical CDS small peptide. **G** Small signaling peptide

Findings revealing the protein-coding capacity of noncoding RNAs have markedly changed the view of small peptides. MicroRNAs constitute a group of small RNAs of approximately 21–24 nt that are processed from pri-miRNA and pre-miRNA and mediate mRNA degradation, translational inhibition, or DNA methylation. Surprisingly, pri-miRNAs can be translated into peptides (miPEPs) ranging from 3 to 59 aa (Lauressergues et al. 2015) (Fig. 1A). The same group recently reported that 84 of 93 pri-miRNAs harbor at least one ORF in the 5′ arm (Lauressergues et al. 2022).

The fact that pri-miRNAs encode small peptides raises the question of whether other noncoding RNAs can encode proteins. As expected, long noncoding RNAs have been reported to be sources of new peptides in *Arabidopsis*, *Physcomitrella patens* (moss), maize and *Glycine max* (soybean), as well as in yeast and animals (Ruiz-Orera et al. 2014; Wang et al. 2020b; Lin et al. 2019). Notably, early nodulin 40 (ENOD 40) has been identified as a long noncoding RNA (lncRNA) in soybean, but this classification has been changed since researchers found that it encodes two small polycistronic peptides (Kageyama et al. 2011). Interestingly, transcripts from the *TAS* loci, precursors of tasiRNA/phasiRNAs, play a role in encoding small peptides (Bazin et al. 2017). Additional evidence has been shown in moss and maize, broadening our knowledge of lncRNAs (Wang et al. 2020b) (Fig. 1B).

Ribosomal RNAs (rRNAs) are the most abundant RNAs in a cell and mediate translation as structural RNAs. However, the 23S rRNA of *Escherichia coli* encodes a functional pentapeptide (Tenson et al. 1996) (Fig. 1C). Furthermore, mitochondrial 16S rRNA encodes a functional peptide that is promising in Alzheimer’s and cancer therapy (Maximov et al. 2002). An inspiring view may reveal the reason that structural rRNA has the ability to encode a protein. Specifically, rRNA has been proposed to be the primary origin of mRNA-encoded ribosomal proteins (Root-Bernstein and Root-Bernstein 2016). Nevertheless, overall evidence of rRNA-encoded peptides is very rare, with no cases in plants. Circular RNAs (circRNAs) are formed by covalent conjunction of the upstream 5′ phosphoryl group and downstream 3′ oxhydryl of a linear transcript and are ubiquitous in a wide range of organisms (Guria et al. 2020). It has been suggested that circRNAs act as miRNA sponges and transcriptional and translational regulators of RNAs, but increasing evidence has shown that circRNAs also encode functional peptides related to different disease models (Fig. 1D) (Zhang et al. 2018; Zheng 2019). Interestingly, translation of circRNAs is cap independent, relying on m^6^A modification at the internal ribosome entry site (Guria et al. 2020). However, no peptide-coding circRNAs have been found in plants to date.

### Small peptides derived from protein-coding genes

Small peptides derived from coding genes can be further grouped into different types according to their relative position in the gene body. Upstream small peptides are translated from the ORF within the 5′ untranslated region (UTR) of the coding gene (uORF) (Fig. 1E). In plants, uORFs account for approximately  20% of transcripts (Nyikó et al. 2009). One of the representatives is the uORF of *TBF1* named uORF_TBF1_, which has been groundbreakingly utilized to regulate translation (Xu et al. 2017a). Downstream small peptides are translated from an ORF in the 3′UTR. Although this type of small peptide is rarely found in plants, it was recently characterized in moss and *Vitis vinifera* L. (grape), including Pp3c23_sORF1096 (Fesenko et al. 2019; Hsu and Benfey 2018; Pei et al. 2022). Genes encoding small peptides are widespread in chloroplasts. More than ten small proteins are parts of photosystem II, such as Psb*E* and Psb*F* subunits, Psb*H*, Psb*I*, and Psb*J*, which are believed to stabilize and assemble to form the PS II complex (Shi and Schröder 2004). Noncanonical CDS small peptides are derived from translated regions between the 5′UTR and 3′UTR, though they can be translated in noncanonical +2 or +3 frames overlapping with the main ORF +1 frame (Fig. 1F) (Hellens et al. 2016; Ong et al. 2022). One example is Pp3c11_sORF461, which is translated accompanied by its main ORF (Fesenko et al. 2019). Short isoforms are translated from genes undergoing alternative splicing as a template (Ong et al. 2022). For instance, an alternative splicing variant of *INDETERMINATE DOMAIN 14* (*IDD14*) encoding IDD14β acts as a small interfering peptide against the full-length peptide (Seo et al. 2011). Another type of plant small peptide derives from a long prepropeptide (a fully encoded mature protein that is subsequently post-translationally processed) (Fig. 1G). Post-translational processing is needed to generate mature functional small peptides (Matsubayashi 2011). These peptides are also called hormone-like peptides because they act as ligands and bind to receptors on the cell membrane in a cell-to-cell manner, resembling the action of hormones, such as CLE, RALF and FER (Takahashi et al. 2019). Some mature proteins require proteolytic cleavage to generate smaller peptides in response to various cellular processes. Interestingly, C-terminal truncated H2A and H2B and N-terminal truncated H3 are found in different cell types (Yi and Kim 2018). Proteolytic cleavage can also be induced by other organisms, as proteolytic cleavage peptides are enriched in Populus root tips after ectomycorrhiza establishment (Chen et al. 2020a; Villalobos Solis et al. 2020). Small peptides called interlaced small peptides span the 5′UTR or 3′UTR and the CDS, and the transcript is the same as that of the main ORF, such as Pp3c12_sORF172 and Pp3c16_sORF2123 in moss (Fesenko et al. 2019).

### Small secretory peptides

Small secretory peptides function outside the cell where they are synthesized and are exported by either the conventional protein secretion pathway (through the Golgi apparatus or trans-Golgi network) or the unconventional protein secretion pathway (directly through the endoplasmic reticulum or multivesicular bodies) (Hu et al. 2021). Most small peptides identified to date are secretory peptides, which are similar to other common secretory proteins and carry leader sequences at the N-terminus. These peptides are transported by the conventional protein secretion pathway. Recent findings suggest that the positive charge in the n-region of the signal peptide is responsible for efficient translocation of secretory peptides (Guo et al. 2018). However, some secretory peptides lack the N-terminal signal sequence and are secreted through excyst-positive organelles (EXPOs) (Hu et al. 2021). Small secretory peptides can be further categorized into post-translationally modified small peptides and cysteine-rich small peptides. The former is processed from prepropeptides and propeptides, undergoing proteolysis and some post-translational modifications on specific residues, including tyrosine, proline and hydroxyproline residues. Some relevant enzymes catalyzing processing and post-translational modification have been verified (Stührwohldt and Schaller 2019; Tabata and Sawa 2014). Peptides that mature in the endoplasmic reticulum or Golgi apparatus usually comprise fewer than 20 aa. Well-known post-translationally modified small peptides in plants include CLEs, PSK, and PSY (Matsubayashi 2014). Cysteine-rich small peptides are thought to undergo several rounds of duplication. They also contain a conserved secretory signal at the N-terminus and an even number of cysteine residues at the C-terminus that is important for intramolecular disulfide bond formation catalyzed by plant disulfide isomerases, which prevents their degradation by proteases (Marshall et al. 2011; Olsson et al. 2019). Due to the diversity of the C-terminus and duplication events, many differences have been observed among subgroups of cysteine-rich small peptides, and obvious differences in gene number have been found between *Arabidopsis* and rice (Marshall et al. 2011). Some well-characterized cysteine-rich small peptides in plants include RAPID ALKALINIZATION FACTOR (RALF), EPF, LURE, PDF, XIUQIU1/2/4, and SP11 (Hu et al. 2018; Matsubayashi 2014; Okuda 2021).

### Nonsecretory small peptides

Nonsecretory small peptides generally lack an N-terminal signal sequence; therefore, they are retained in the cell. They include two types of peptides: transmembrane peptides that mediate cellular communications and membrane architecture and intracellular peptides that function as regulators in the signaling network (Xu et al. 2017c). However, in some cases, nonsecretory small peptides can be released from the cell and transmit cell-to-cell signals in response to damage. Systemin and AtPep1 share the above-mentioned characteristics and are considered to be extracellular nonsecretory peptides (Pearce and Ryan 2003; Pearce et al. 2008; Ryan and Pearce 1998). Recently, a novel type of nonsecreted small peptide termed the cysteine-rich transmembrane module (CYSTM) was characterized. As its name implies, this type of peptide is enriched with cysteines and is, therefore, also called a nonsecreted cysteine-rich peptide (NCRP). Interestingly, cytoplasmic or plasma membrane-localized NCRPs undergo homodimerization or heterodimerization (Xu et al. 2017c). Plant defensin AtPDF2.6 localizes to the cytoplasm, though it is predicted to be secreted in accordance with its signal peptide (Luo et al. 2019a); this finding highlights the importance of experimental verification in concluding the secretory capability of small peptides. In addition, most small peptides encoded by noncoding RNAs functioning inside cells are not secreted, such as uORFs and miPEPs, which modulate translation and transcription (Nelde et al. 2022; Prasad et al. 2021). Notably, ENOD40, previously regarded as a noncoding gene, has been validated to function as a nonsecretory small peptide (Scheidler et al. 2019). Other previously reported intracellular nonsecretory small peptides include POLARIS (PLS) and ROTUNDIFOLIA 4 (ROT4)/DEVIL (DVL16) (Gancheva et al. 2019; Luo et al. 2019b).

## Evolutionary insight into plant small peptides

Conservation analysis of small peptides, which has been widely employed in identifying protein-coding genes, is a possible strategy to identify functional small peptides. Since conservation of lncRNAs is low among plant species (Deng et al. 2018), it is reasonable that small peptides derived from lncRNAs have also diverged. One analysis identified more than 5000 conserved sORFs among 10 plant species using public transcriptome data, but only five of these identifications were supported by MS data, among which three sORFs are located in lncRNAs (Fesenko et al. 2019).

Small peptides encoded by protein-coding genes originate from various hierarchies during evolution. The ancient small peptide families CLE, IDA and CIF ascended in hornworts. RGF, PSK and PSY appeared later in liverworts and mosses. Others belong to the TWS1, CEP, PIP and PEP families, which evolved after vascular plants (Furumizu et al. 2021). Notably, our knowledge about the conservation of small peptides should not be limited to their primary sequences, but proteolytic processing as well as post-translational modifications should be taken into consideration (Furumizu et al. 2021).

Interestingly, miPEPs from closely related plant species vary in sequence composition, even though the number of ORFs has been maintained. Nonetheless, nonconserved miPEPs can execute conserved functions by activating transcription of corresponding pri-miRNAs after recognizing the miORF. When changing the peptide sequence of a miPEP, activation of pri-miRNA expression will be abolished (Lauressergues et al. 2022).

## Biological functions of plant small peptides

Plant small peptides have been found to function in various aspects of growth and development, suggesting that they are potential resources for agricultural regulators. Biological functions of plant small peptides include abiotic stress response, root growth, leaf morphogenesis, and plant reproduction.

### Response to abiotic factors

Plant small peptides are reported to respond to different abiotic stresses. Overexpression of a 10-aa small peptide-coding gene, *OSIP108,* confers tolerance to paraquat, which causes oxidative stress, in *Arabidopsis* (De Coninck et al. 2013). CLE25 was discovered to move from roots to leaves during drought stress and induce abscisic acid (ABA) accumulation (Takahashi et al. 2019). Small peptides have also been identified in the response to other abiotic stresses, such as exposure to flooding or high salinity (Nakaminami et al. 2018; Nanjo et al. 2011), indicating that small peptides are widely involved in responses to various abiotic stresses. Recently, two small peptides, miP1a and miP1b, were discovered to modulate photomorphogenesis in response to light by disassociating the PIF-EIN3 complex, revealing novel functions of small peptides (Wu et al. 2020). Nutrient deficiency is a stress caused by environmental factors, and small peptides help plants cope with such conditions. For example, phloem-expressed FEP/IMA responds to iron deficiency and promotes iron uptake, and root-expressed CEP modulates signaling during low-nitrogen conditions (Grillet et al. 2018; Hirayama et al. 2018; Okamoto et al. 2016).

### Disease resistance

Small peptides function in host defense against bacteria, fungi, viruses, and pests. Cyclotides are cyclic peptides comprising approximately 40 residues and are produced in several dicot plant families (Pan et al. 2019). Evidence has shown that certain types of cyclotides play a role in antibacterial immunity (Habbadi et al. 2017; Panneerselvam et al. 2015). The secreted peptide ROOT MERISTEM GROWTH FACTOR 7 (RGF7) is induced by *Pseudomonas syringae* to elicit immunity in *Arabidopsis* (Wang et al. 2021). In response to fungal attack, *Medicago sativa* and *Pharbitis nil* generate alfAFP and Pn-AMP2 peptides to defend against *Verticillium dahliae* infection (Pan et al. 2019). Although few small peptides involved in antiviral immunity have been identified, PAMP-induced peptide 1 (StPIP1), either exogenously added or overexpressed**,** was recently found to confer resistance to potato virus Y (Combest et al. 2021). Some small peptides also play negative roles in virus infection. Virus-induced small peptide 1 (VISP1) interacts with ATG8 and SGS3 to induce autophagy, which compromises antiviral immunity by attenuating the RNA interference pathway (Tong et al. 2021). Cyclotides are reported to play a role in the response to invading insects. For example, cyclotide Kalata B1 inhibited the growth of *Helicoverpa punctigera* larvae by destroying their midgut membrane (Barbole et al. 2022). In addition, many growth-related small peptides have been shown to modulate plant immunity by fine-tuning pathways: plant elicitor peptides (PEPs), PIPs and serine-rich endogenous peptides (Scoops) amplify immune; cysteine-rich secretory proteins, antigen 5, and pathogenesis-related 1 protein-derived peptides (CAPEs) enhance the function of their precursor PR1; and 1 the 7-aa Zip1 promotes SA-dependent immunity. In addition, RALFs have been shown to affect the functions of their receptors, and PSK and PSY play various roles in response to different pathogens (Segonzac and Monaghan 2019).

### Root growth and development

miPEP171b in *M. truncatula* and miPEP165 in *Arabidopsis* influence lateral root density and root length, respectively, by inducing expression of their pri-miRNAs (Lauressergues et al. 2015). Moreover, a 36-aa small peptide PLS has been found to regulate root length by affecting cell expansion (Casson et al. 2002). Other small peptides have been reported to regulate different aspects of root growth and development. In the meristematic zone, CLE17, CLE19 and CLE40 regulate stem cell fate (Zhu et al. 2020). In the elongation zone, the TYROSYL-PROTEIN SULFOTRANSFERASE (TPST)-derived post-translationally modified small peptides PSK and PSY affect cell elongation. In the differentiation zone, CASPARIAN STRIP INTEGRITY FACTORS (CIF) and RALF function in Casparian strip formation, lateral root initiation and root hair growth (Hsiao and Yamada 2021). Interestingly, CLE peptides function as negative regulators, but other peptides function as positive regulators in root growth and development, revealing the functional diversity of small peptides.

### Leaf morphogenesis

Several small peptides are involved in morphogenesis. PLS, in addition to affecting root length, is critical for normal leaf vascular patterning. Semidominant mutants of PLS display reduced vascularization (Casson et al. 2002). The 53-aa ROTUNDIFOLIA4 (ROT4) protein is located in the leaf cell membrane and affects leaf size and shape (Narita et al. 2004). The 76-aa small peptide Brick1 (Brk1) is conserved in both plants and animals, and mutation of the gene in maize results in a disrupted epithelial pattern due to impaired organization of the cell skeleton (Frank and Smith 2002).

### Organ size determination

Organ size is an important trait in agriculture. A recent study revealed that an unannotated gene encoding the 57-aa small peptide AtZSP1, which resides in the endomembrane, positively regulates organ size by promoting endogenous cytokinin levels (Zeng et al. 2022). Interestingly, homologues of AtZSP1 have been found in soybean, maize, *Setaria italica* (Foxtail millet), and *Brassica rapa* (Chinese cabbage), indicating a conserved function for this small peptide in plants (Orr et al. 2020). In contrast, the nonsecreted small peptide SPICE negatively affects pistil size by mediating cell expansion in tobacco (Brito et al. 2018).

### Crop domestication

Domestication confers multiple advantageous traits to modern cultivars. Awn is a significant trait associated with domestication; the structure is obvious in ancient rice cultivars but has degenerated in cultivated rice. A small peptide RAE2/GAD1 belonging to the EPIDERMALPATTERNING FACTOR-LIKE (EPFL) family plays a role in awn elongation. Mutation leading to a truncated mature RAE2/GAD1 peptide in Asian rice loses its functions and causes an awnless phenotype (Bessho-Uehara et al. 2016). Moreover, mutations of RAE2/GAD1 lead to shorter grain length and increased grain number per panicle, resembling cultivated traits (Jin et al. 2016). Another member of this family, OsEPFL2, is critical for awn development in Kasalath. Further study on sequence polymorphisms has revealed that OsEPFL2 underwent positive selection during domestication (Xiong et al. 2022). The relationship between small peptides and domestication in other plant species is not clear, yet a large-scale analysis has suggested that 55 of 1993 identified small peptides in maize are related to domestication selection; the functions related to these peptides remain to be determined (Wang et al. 2020b).

### Plant reproduction

Many small peptides, especially hormone-like peptides, play regulatory roles in various stages of sexual reproduction. The CLV3 rice orthologue OsFON2 has been reported to reduce floral size and number. Nevertheless, these orthologues in maize and *Setaria viridis* play different roles. Inflorescence meristem size but not floral meristem size is altered in *svfon2* mutants and *zmcle7* and *zmcle14* mutants, suggesting specific functions of CLE peptides in panicoid plants (Zhu et al. 2021). A phytosulfokine family member in *Pyrus bretschneideri*, PbrPSK2, promotes pollen tube growth and germination when exogenously applied to pear pollen. Further experiments showed that the growth-promoting effect was caused by elevated reactive oxygen species production (Kou et al. 2020). Pollen-localized S-locus Cys-rich/S-locus protein 11 (SCR/SP11) recognizes small peptide receptor kinase (SRK) to modulate self-incompatibility in *Brassica napus* (Samuel et al. 2009). Furthermore, PCP-Bα/β/γ/δ in *Arabidopsis*, LAT52 in tomato, and Zm908p11 in maize are critical for pollen germination and pollen tube growth (Dong et al. 2013; Tang et al. 2002; Wang et al. 2017). AtLURE1 expressed in egg-accompanying synergid cells is secreted and involved in pollen tube attraction to facilitate conspecific fertilization (Zhong et al. 2019). However, some small peptides, such as ZmES1-4, function in pollen tube reception to stop pollen tube growth and promote sperm release (Rotman et al. 2003). Gamete activation is an important process after sperm release. EGG CELL 1 (EC1) is necessary for this process because it rearranges potential fusogen in the inner membrane system of sperm (Sprunck et al. 2012). As the currently identified small peptides known to participate in sexual reproduction are derived from protein-coding genes, it is unclear whether small peptides from noncoding genes are also involved in sexual reproduction.

## Mechanisms of small peptides in plant regulatory networks

Investigation of the molecular mechanisms of plant small peptides may help scientists to better improve crop traits or utilize peptides in biotechnological applications. The molecular mechanisms of small peptides in plants known to date include translational inhibition of a main ORF (mORF), promotion of miRNA function, signal transduction and interplay with target proteins.

### Promoting functions of miRNAs

**Fig. 2:**
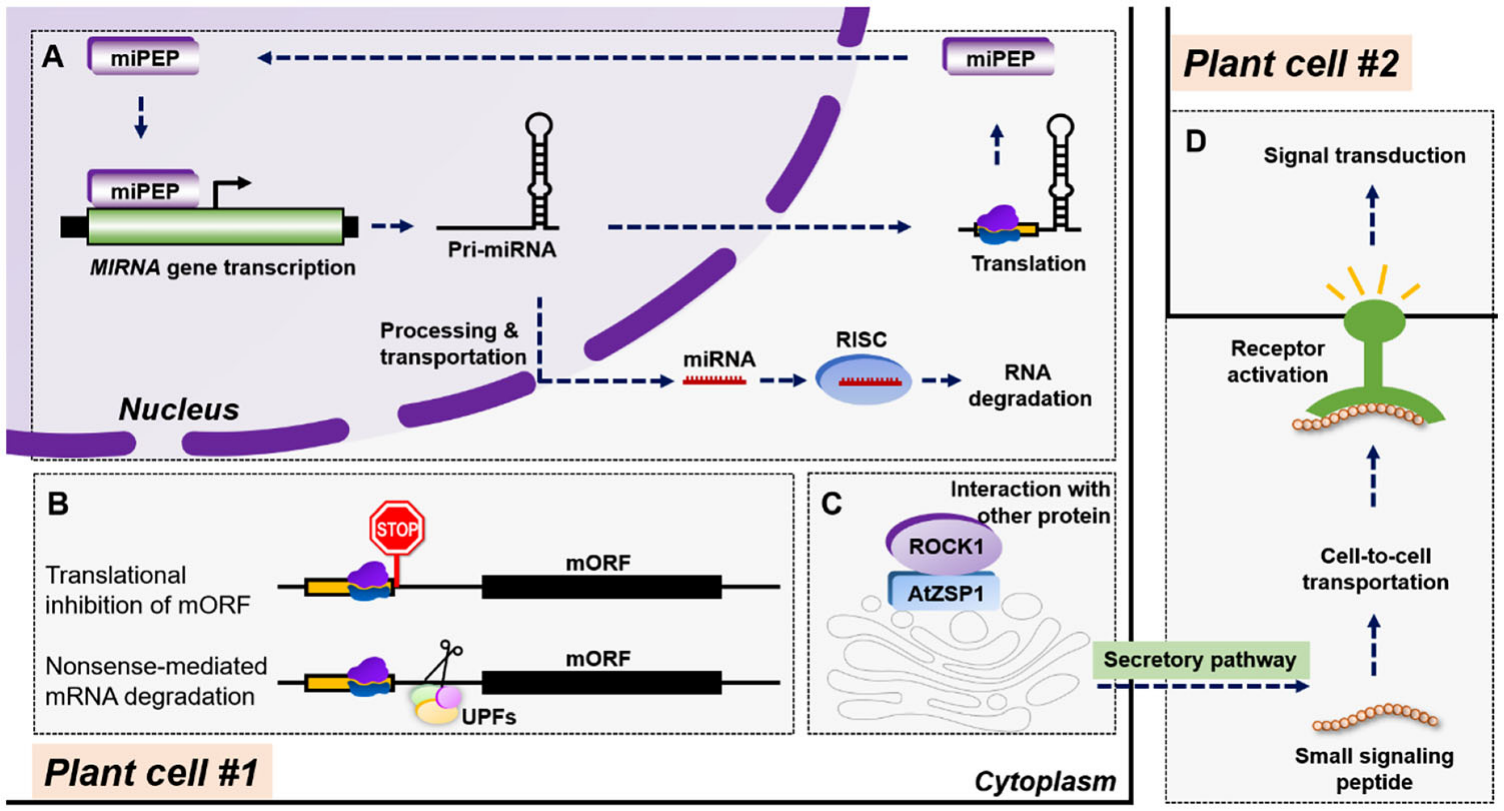
Molecular mechanisms of plant small peptides. **A** MiPEPs regulating MIRNA transcription. **B** UPFs mediating mORF protein abundance translationally and post-transcriptionally. **C** Small peptides interacting with other proteins within a cell. **D** Small secretory peptides binding to receptors for signal transduction

miPEPs are small peptides that exert a miRNA-regulating effect. Lauressergues and colleagues discovered that miPEP171b in *Medicago truncatula* and miPEP165a in *Arabidopsis* elevate the transcriptional level of the pri-miRNA from which they derive and thereby downregulate target genes (Lauressergues et al. 2022). Similarly, other miRNAs are induced by corresponding miPEPs, indicating a conserved regulatory mode of action (Lauressergues et al. 2022) (Fig. 2A). Considering this feature, scientists have used certain miPEPs to modulate crop traits. For example, miPEP172c has been used to regulate nodulation in soybean because it increases accumulation of miR172c. MiPEP858a regulates flavonoid biosynthesis in *Arabidopsis* because it increases accumulation of miR858a (Couzigou et al. 2016; Sharma et al. 2020). Further studies have shown that miPEPs can activate only their own pri-miRNAs. Therefore, the functional specificity of a miPEP depends on its location and coding sequence within the pri-miRNA sequence. Förster resonance energy transfer-fluorescence lifetime imaging microscopy (FRET-FLIM) and isothermal titration calorimetry (ITC) experiments support the idea that miPEP directly interacts with the corresponding miORF in RNA (Lauressergues et al. 2022).

### Translational inhibition of mORF

uORF-encoded peptides have been reported to attenuate or inhibit translation of downstream mORFs. When an upstream small peptide is translated, ribosome scanning on the mORF is inhibited; in some cases, ribosomes dissociate from mRNA when they reach the stop codon of the small peptide and thus trigger nonsense mRNA decay (NMD) resulting from ribosome stalling (Fig. 2B) (Zhang et al. 2020). One of the applications of this characteristic involves engineering *TBF1p::uORF*_*TBF1*_*-AtNPR1* in rice, conferring disease resistance without inducing fitness penalties (Xu et al. 2017b).

### Interplay with other proteins

Some small peptides are located in specific organelles and thus may interact with other proteins in that compartment. For example, on the basis of its mutant phenotype, the endoplasmic reticulum (ER)-localized small peptide TWS1 has been assumed to interact with proteins resident in the ER or Golgi (Fiume et al. 2016). Small peptides may also change the conformation of a target protein and facilitate its own interaction with other proteins. Interaction between small peptides and other proteins may lead to a negative effect when they block the functional domain of a target protein (Storz et al. 2014). The endomembrane-associated small peptide AtZSP1 also interacts with the ER-localized UDP-GlcNAc/UDP-GalNAc transporter ROCK1 to affect organ size (Zeng et al. 2022) (Fig. 2C). These examples suggest that small peptides interact with other proteins. In general, identification of proteins that interact with small peptides may lead to a better understanding of the mechanisms of small peptide action.

### Signal transduction

A class of small peptides are excreted outside the cell, functioning as ligands that induce signal transduction (Fig. 2D). CLAVATA3/EMBRYO-SURROUNDING REGION-RELATED (CLE) family member CLE25 originates in the root vasculature and is transported to leaves, where it binds its receptors BARELY ANY MERISTEM (BAM) 1 and BAM3 in response to osmotic stress (Takahashi et al. 2019). Another member of the same gene family, CLE45, binds its receptors STERILITY-REGULATING KINASE MEMBER1 (SKM1) and SKM2, which belong to the leucine-rich repeat receptor-like kinase (LRR-RLK) family, on the surface of pollen tubes (Zhang et al. 2021). CLE9/10 binds to another LRR-RLK member, HSL1, the action of which is promoted in the presence of SOMATIC EMBRYOGENESIS RECEPTOR-LIKE KINASE (SERK) coreceptors SERK1, SERK2, and SERK3/BRASSINOSTEROID INSENSITIVE1-ASSOCIATED RECEPTOR KINASE1 (BAK1) (Takahashi et al. 2019). The receptors of some small peptides are currently unknown, and the underlying mechanisms of action for other small proteins are not understood, though some are thought to function in important signaling pathways; for example, CAPEs and Zip1 are involved in SA-mediated immunity (Chen et al. 2014; Ziemann et al. 2018).

## Experimental strategies for studying plant small peptides

Although various types of plant small peptides have been discovered, characterization of small peptides is still difficult mainly because small peptides are too small to be characterized. In the past, computational programs have tended to eliminate ORFs less than 300 nt long; moreover, the criteria for identifying ORFs included translation start and stop signals, splice signals, and polyadenylation signals, among others, which hindered classification of small peptides (Ong et al. 2022). However, with the development of bioinformatics, biochemical and molecular techniques, it is becoming increasingly possible to validate the identity of previously ignored plant small peptides.

### Genome-wide mining for novel small peptides

**Fig. 3:**
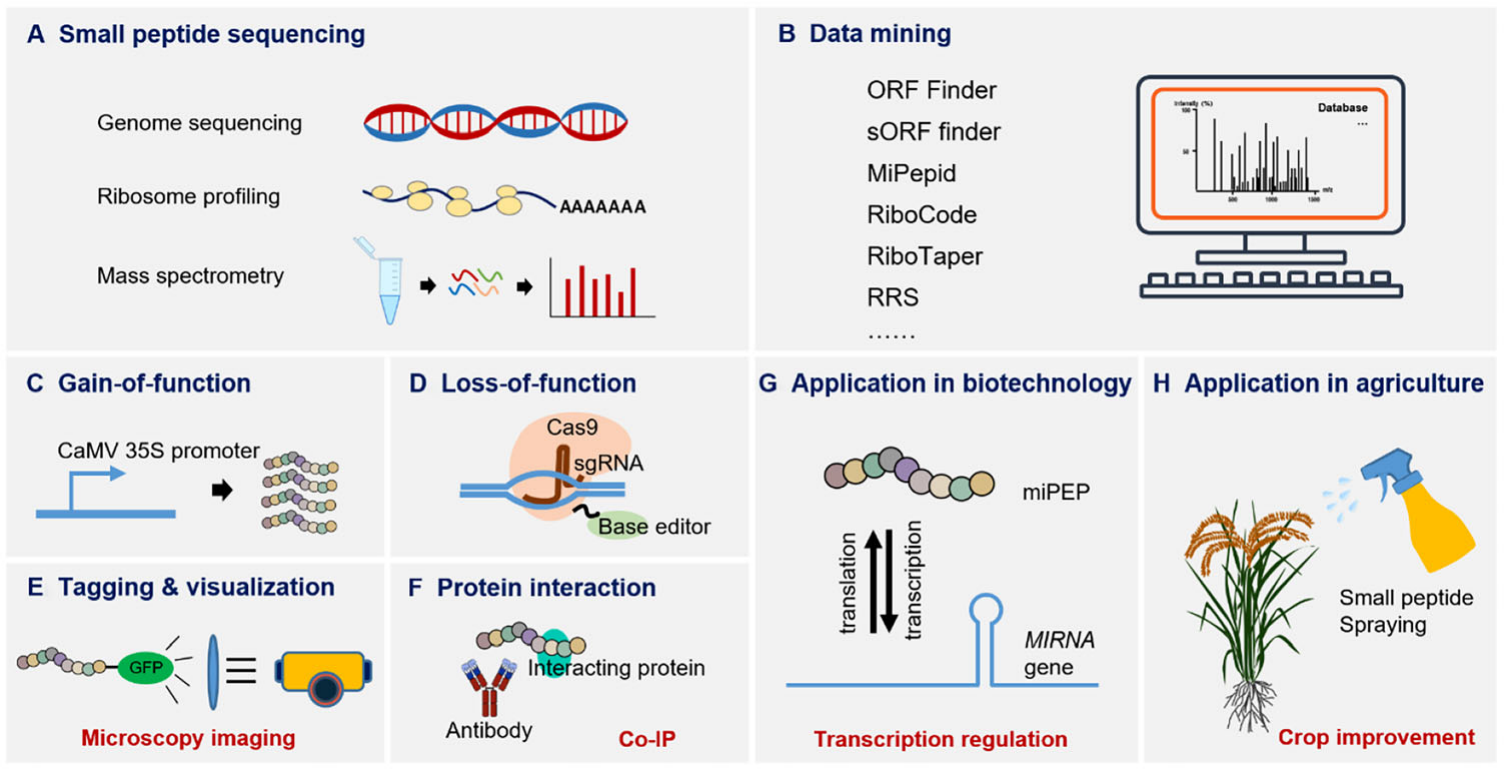
Strategies and applications of studying plant small peptides. **A** Discovery of small peptides by multi-omics. **B** Analysis of high-throughput data by computational programs. **C**–**F** Biochemical and molecular studies by overexpression (**C**), loss-of-function (**D**), subcellular localization (**E**) and interacting protein identification (**F**). **G**, **H** Applications of small peptides in biotechnology research (**G**) and agriculture (**H**)

Not all ORFs encode peptides, but many computational tools can help in predicting small peptides based on the following criteria: sequence composition, putative peptide length, degree of peptide conservation, protein similarity and domain similarity (Housman and Ulitsky 2016). Basic Local Alignment Search Tool (BLAST) and ORF Finder are used to predict peptides by referring to sequence homology and have been used to predict small peptides in drosophilids, *Populus deltoides*, and *Populus trichocarpa* (Magny et al. 2013; Mewalal et al. 2019). Other tools, such as PhyloCSF and uPEPperoni, have been employed for predicting small peptides according to the degree of phylogenetic conservation of peptide-coding sequences and have been specifically trained for small peptide prediction, similar to ANNOgesic, MiPepid, and sORF finder (Hanada et al. 2007; Ong et al. 2022; Schlesinger and Elsässer 2022; Yu 2019; Zhu and Gribskov 2019) (see Fig. 3).

Genome-wide identification of small peptides in several species has been performed. For example, Lin et al. (2019) identified 153 unique small peptides encoded by soybean lncRNAs, contributing to functional investigation of these lncRNAs. In addition, Liang et al. (2021) characterized 2695 small peptides in six tissues of maize using multi-omics data. Some studies have aimed to identify small peptide functions in certain processes, leading to identification of small peptides via specific treatment. Wang et al. (2020a) discovered 52 novel small peptides involved in immunity in rice using chitin as an elicitor. In an innovative study, Ohyama et al. (2008) developed an efficient system to identify secreted peptides. Specifically, the authors extracted peptides from the medium of a whole plant in submerged culture using o-chlorophenol and detected an uncharacterized small, secreted peptide gene family in *Arabidopsis*. Other research teams have focused on specific small peptide families in noncereal crops; for instance, genome-wide analyses have led to identification of CLE in *Malus* × *domestica* (apple), the GASA family in *Cucumis sativus* (cucumber), and the defensin family in *Arachis hypogaea* L. (peanut) (Zhang et al. 2022a, 2022b; Zhao et al. 2022).

### Ribosome profiling

Ribosome profiling (Ribo-seq) is a powerful method to identify proteins involved in translation: ribosomes binding to mRNAs are captured, after which the ribosome-protected RNAs are recovered and subjected to high-throughput sequencing. More ribosomes that bind to RNA indicate a higher likelihood of translation. However, this approach may lead to false-positive results because some folded RNAs will not be digested by a nuclease for determination by Ribo-seq (Brar and Weissman 2015; Guttman et al. 2013). In addition, some RNAs bound to ribosomes, such as lincRNA-p21, AS-RBM15, and AS-Uchl1, are not translated into proteins (Carrieri et al. 2012; Tran et al. 2016; Yoon et al. 2012). Guttman et al. stated that ribosome occupancy is not a fully reliable determinant of translation and proposed a metric based on judging ribosome release at a stop codon (Guttman et al. 2013). Some small peptides are not stable, and these small peptides are regarded as mere by-products. Therefore, many computational tools can be used to further filter Ribo-seq data, and all of these tools were developed in the past decade (Bazzini et al. 2014; Calviello et al. 2016; Chew et al. 2013; Chun et al. 2016; Erhard et al. 2018; Fields et al. 2015; Guttman et al. 2013; Ingolia et al. 2014; Ji et al. 2015; Malone et al. 2017; Michel et al. 2012; Raj et al. 2016; Ruiz-Orera et al. 2014; Song et al. 2021; Xiao et al. 2018; Xu et al. 2018; Zhang et al. 2017). Among these tools, RiboCode, RiboTaper, RRS, translation efficiency (TE), and RiboNT have successfully been applied for identifying plant small peptides. Researchers have also searched for small peptides in databases (Chen et al. 2020c; van Wijk et al. 2021). The best way to confirm the identity of small peptides is via MS analysis, which reveals the amino acid composition and abundance in vivo.

### Mass spectrometry

Mass spectrometry is a credible method to identify and quantify proteins and is a more direct measure than RNA-based ribosome or polysome profiling. Detailed information such as protein isoforms and post-translational modifications can be acquired through this technique. However, due to the special properties of small peptides, the pipelines for identification based on mass spectrometry need to be modified. Important suggestions have been put forth by Ahrens et al. including removal of cutoff filters, reduced washing stringency, lowered organic solvent concentrations during sample preparation, use of proper enzymes for protein digestion, adjustments to the gradient length on the basis of sample complexity liquid chromatography and use of matrix-assisted laser desorption ionization (MALDI) (Ahrens et al. 2022). In general, using standard databases to analyze the spectrometry results may result in underrepresentation of candidates or false-positive hits, and custom databases are needed to compare sequences in six frames (Orr et al. 2020). Small peptides generate only a single spectrum, which makes a signal difficult to detect or interpret (Murray et al. 2018; Orr et al. 2020), and dimethyl labeling has been applied to enhance the signal of a_1_ ions, improving small peptide identification in almonds. Studying the mass spectrometry characteristics of small peptides may help in identifying unknown peptides in the same family, and a synthesized peptide may be used to verify newly discovered small peptides (Ahrens et al. 2022; Yuan et al. 2022).

### Genetic approaches

To investigate gene functions, biologists often create mutants of a target gene. This strategy can also be applied to study plant small peptides. Genome editing targeting an initiation codon or a coding sequence of a small peptide can be applied to obtain loss-of-function mutants (Fesenko et al. 2019; Sharma et al. 2020; Sousa and Farkas 2018). However, even obvious morphological changes cannot always be easily seen, probably due to the redundancy or weak effect of a small peptide. Alternatively, overexpression of small peptides can provide additional insight into their functions. Hanada et al. overexpressed 473 small peptides in Arabidopsis and observed the phenotypes of 49 mutants overexpressing small peptides (Hanada et al. 2013). Nevertheless, constitutive overexpression may lead to acquisition of unexpected phenotypes due to abnormal alteration of key pathways via a sudden increase in a transgenic protein throughout all tissues, which may interfere with accurate evaluation of small peptide functions. Moreover, strong expression of a gene homologous to an endogenous gene may trigger cosuppression effects, which may also mask the true roles of a small peptide. Alternatively, expressing a small peptide driven by a native promoter may be a useful way to study small peptide functions via genetic approaches.

### Tagging and visualization

Western blotting is a common technique used to assess protein expression. However, preparation of antibodies for a peptide is time-consuming and costly, and the specificity and efficiency of an antibody are typically uncertain. Therefore, tags can be fused in-frame with small peptides to determine their expression. CRISPR–Cas9-mediated genome editing may help with inserting FLAG/His/HA tags, thereby facilitating protein detection with a commercial tag-specific antibody instead of an antibody specifically targeted to the small peptide of interest (Yeasmin et al. 2018). Furthermore, using a GFP tag and fluorescence microscopy, researchers can view the subcellular localization of small peptides, aiding in characterization of their function (Pauli et al. 2014). However, the localization or function of small peptides might be disrupted when a tag is too large relative to the size of the small peptide (Ong et al. 2022).

### In vitro translation and chemical synthesis of plant small peptides

Cell-free translation is a time-saving method to determine whether a small peptide can be translated. This method requires the combination of a T7 or SP6 promoter and the full-length cDNA of a small peptide, which are needed to produce mRNA. Next, under the conditions dictated by translation machinery, substrates, and energy sources, nascent peptides are identified via incorporation of radio-labeled ^35^S-methionine (Ong et al. 2022; Sousa and Farkas 2018). Chemical synthesis ensures high purity and desired labeling of small peptides, providing an effective tool for studying plant small peptide functions. Owing to their small size, small peptides can be absorbed by roots and transported to other parts of the plant. Thus, researchers can observe molecular and morphological effects by applying exogenous small peptides to plants (Erokhina et al. 2021; Lauressergues et al. 2015, 2022). More importantly, because small peptides originate from plants, they are safe for regulating crop growth without causing environmental pollution.

## Application of small peptides in biotechnology and agriculture

Their low molecular weight and small size render small peptides ideal chemicals for exogenous applications in agriculture. Synthesized miPEP172c has been applied to promote nodule growth in soybean (Couzigou et al. 2016), and synthetic miPEP-156a from Napa cabbage promotes primary root growth in cabbage seedlings (Erokhina et al. 2021). miPEP modulates a secondary metabolism pathway. Synthetic miPEP164c has been added to grape berry cell culture, where it markedly increased the anthocyanin content (Vale et al. 2021). MiPEP is also a good tool for laboratory work because it can facilitate the study of miRNA function in nonmodel plants for which genetic transformation is not possible and prevent acquisition of artificial phenotypes induced by ectopic expression (Couzigou et al. 2015).

Moreover, small peptides can be used to enhance plant defenses, as some small peptides are protease inhibitors or antimicrobial agents that attack pathogens and pests. For example, *Brassica rapa* Defensin1 expressed in rice promotes immunity against brown planthopper (Choi et al. 2009). Importantly, small peptides that are helpful in disease therapy can be largely produced via plant-based expression systems (Barbole et al. 2022; Holaskova et al. 2015), such as peptides in chickpea capable of decreasing total cholesterol, triglyceride, and low-density lipoprotein cholesterol and increasing high-density lipoprotein cholesterol (HDL-C) content in serum. Using tissue-specific promoters to express peptides limits side effects in other parts of a plant and the environment, such as prolamin and glutamin promoters in rice (Holaskova et al. 2015). Furthermore, an exciting study showed that applying complementary peptides (cPEPs) can increase the expression level of their target protein and thus enhance biological function. Tomato treated with cPEPjar1 displays enhanced resistance to *B. cinerea*. A cocktail of cPEPs targeting multiple proteins is also capable of controlling weed growth in *B. vulgaris* and *A. hypochondriacus*, which greatly expands the application of small peptides in biological control (Ormancey et al. 2023). More interestingly, small peptides from animals show bioactivity in regulating expression of plant small peptide CLE family genes (Fedoreyeva et al. 2017). As small peptides exhibit multiple functions in stress responses, growth and development, future applications focusing on those aspects are expected.

## Conclusion and perspectives

The development of computational prediction and ribosome profiling tools, together with mass spectrometry, has led to great advances in discovering small peptides in plant genomes (Table 1). Small peptides are categorized according to their genetic origin as protein-coding/noncoding gene-derived small peptides or according to their distribution as secretory/nonsecretary peptides. Based on emerging research, these molecules play a role in many biological processes, including response to abiotic stress, root growth, leaf morphogenesis, crop domestication, and plant reproduction. The molecular mechanisms of small peptides in plants are currently thought to involve signal transduction, stimulation of miRNA function, translational inhibition, and interaction with target proteins. In addition, a number of examples of small peptides successfully being used in biotechnology and agriculture have been documented in recent years, with many of them having extensive potential applications in crop breeding and improvement.

**Table 1:** Summary of plant small peptides that are functionally characterized

Species	Small peptide	Subcellular localization	Receptor	Function	Reference
*Arabidopsis*	miPEP165	Cytoplasm	–	Root development	(Lauressergues et al. 2015; Xu et al. 2017b)
*Arabidopsis*	miPEP858a	Cytoplasm	–	Flavonoid biosynthesis	(Sharma et al. 2020)
*Arabidopsis*	POLARIS	Endoplasmic reticulum	–	Root growth, vascular patterning, auxin transport, ethylene signaling	(Casson et al. 2002; Chilley et al. 2006; Liu et al. 2013; Mudge 2016)
*Arabidopsis*	ProPeps/Peps	Cytoplasm, plasma membrane, extracellular	LRR-RLKs	Defense against pathogen	(Bartels and Boller 2015)
*Arabidopsis*	TWS1	Endoplasmic reticulum	–	Seed and plant development	(Fiume et al. 2016)
*Arabidopsis*	VISP1	Cytoplasm	–	Defense against pathogen	(Tong et al. 2021)
*Arabidopsis*	mip1a/b	Nucleus	–	Photomorphogenesis	(Wu et al. 2020)
*Arabidopsis*	AtZSP1	Endomembrane	–	Organ size regulation	(Zeng et al. 2022)
*Arabidopsis*	CYSTM	Plasma membrane, cytoplasm, nucleus	–	Environmental stress response	(Xu et al. 2017c)
*Arabidopsis*	ROT4/DVL16	Plasma membrane	–	Polar cell proliferation, leaf morphogenesis	(Ikeuchi et al. 2011; Narita et al. 2004)
*Arabidopsis*	Brk1	Plasma membrane	–	Leaf patterning	(Dyachok et al. 2008; Frank and Smith 2002)
*Arabidopsis*	PSY	Extracellular	PSYR	Cell proliferation, cell expansion, immune response	(Mosher et al. 2013; Tost et al. 2021)
*Arabidopsis*	RALF	Extracellular	FERONIA	Cell division and elongation, pollen tube reception	(Escobar-Restrepo et al. 2007; Haruta et al. 2014)
*Arabidopsis*	LURE	Extracellular	PRK	Ovule attraction	(Takeuchi and Higashiyama 2016)
*Arabidopsis*	CEPs	Extracellular	–	Flower and leaf development, root growth	(Ohyama et al. 2008; Roberts et al. 2013)
*Arabidopsis*	OSIP108	Extracellular	–	Oxidative stress	(De Coninck et al. 2013)
*Arabidopsis*	CIF	Extracellular	GSO1/SGN3 GSO2	Casparian strip formation	(Doblas et al. 2017)
*Arabidopsis*	Scoops	Extracellular	LRR-RLKs	Defense against pathogen	(Gully et al. 2019; Hou et al. 2021)
*Arabidopsis*	PCP-Bα/β/γ/δ	Extracellular	–	Pollen germination	(Wang et al. 2017)
*Arabidopsis*	LTP1	Extracellular	Cryptogein	Somatic embryogenesis, ethylene response	(Buhot et al. 2001; Potocka et al. 2012; Wang et al. 2016)
*Arabidopsis*	GLV/RGF/CLEL	Extracellular	RGIs	Root gravitropic responses, RAM homeostasis, cell divisions, defense against pathogen	(Fernandez et al. 2015; Matsuzaki et al. 2010; Wang et al. 2021; Whitford et al. 2012)
*Arabidopsis*	EC1	Extracellular	–	Gamete activation	(Sprunck et al. 2012)
*Arabidopsis*, pear	PSK	Plasma membrane	PSKR1, PSKR2	Cell proliferation, immune response, pollen tube growth, germination	(Kou et al. 2020; Mosher and Kemmerling 2013; Rodiuc et al. 2016; Sauter 2015)
*Arabidopsis*, potato	prePIPs/PIPs	Extracellular	LRR-RLKs	Defense against pathogen	(Combest et al. 2021)
*Arabidopsis*, rice	EPF/EPFL	Extracellular	ERECTA family receptor kinase	Stoma development, awn development	(Hara et al. 2009; Xiong et al. 2022)
*Arabidopsis*, rice	FEP/IMA	Nucleus, cytoplasm	–	Iron uptake	(Grillet et al. 2018; Peng et al. 2022)
Brassicaceae	miPEP-156a	Cytoplasm	–	Primary root growth	(Erokhina et al. 2021)
Rapeseed	SCR/SP11	Extracellular	SRK	Self-incompatibility	(Shiba et al. 2001)
Soybean	ENOD40	Cytoplasm	–	Nodule formation	(Imaizumi-Anraku et al. 2000)
Soybean	miPEP172c	Cytoplasm	–	Nodulation	(Couzigou et al. 2016)
Tomato	Systemin	Cytoplasm	SYR1	Response to damage	(Wang et al. 2018)
Tomato	CAPEs	Extracellular	–	Defense against pathogen	(Chen et al. 2014)
Tomato	LAT52	Extracellular	LePRK2	Pollen germination	(Tang et al. 2002)
Tobacco	SPICE	Cytoplasm/plasma membrane	–	Organ size regulation	(Brito et al. 2018)
Barrel clover	miPEP171b	Cytoplasm	–	Root development	(Lauressergues et al. 2015)
Alfalfa	alfAFP	Extracellular	–	Defense against pathogen	(Gao et al. 2000; Pan et al. 2019)
Barrelclover	NCR169	Extracellular	–	Symbiotic nitrogen fixation	(Horváth et al. 2015)
Morning glory	Pn-AMP2	Extracellular		Defense against pathogen	(Pan et al. 2019)
Grape berry	miPEP164c	Cytoplasm	–	Anthocyanin biosynthesis	(Vale et al. 2021)
Maize	Zm908p11	Cytoplasm	–	Pollen tube growth	(Dong et al. 2013)
Maize	Zip1	Extracellular	–	Defense against pathogen	(Ziemann et al. 2018)
Maize	ZmES1-4	Extracellular	–	Pollen tube burst	(Amien et al. 2010)
Rice	DEFs	Extracellular	–	Defense against pathogen	(Weerawanich et al. 2018)
Rice	OsDT11	Extracellular	–	Drought tolerance	(Li et al. 2017)
Rice	OsDSSR1	Nucleus and cytoplasm	–	Drought tolerance	(Cui et al. 2018)
Rice	IRP1	Extracellular	–	Defense against pathogen	(Wang et al. 2022)
Rice	OsCDT3	Plasma membrane	–	Aluminum tolerance	(Xia et al. 2013)
*Oldenlandia affinis* (R&S) DC	Cyclotides	Vacuole	–	Defense against pathogen and herbivore	(Conlan et al. 2011; Pan et al. 2019)
Conserved (*Arabidopsis*, rice, etc.)	uORFs	Cytoplasm	–	Attenuate translation of mORF, Defense against pathogen, phosphate acquisition, auxin response, flowering time, etc.	(Guo et al. 2022; Liu et al. 2021; Uzair et al. 2021; Xu et al. 2017b)
Conserved (*Arabidopsis*, rice, etc.)	CLEs	Nucleus, plasma membrane	CLV1, CLV2, CRN, RPK2	Shoot apical meristem size regulation, pollen tube growth, stress response	(Clark et al. 1995; Takahashi et al. 2019; Wan et al. 2021; Zhang et al. 2021)
Conserved (*Arabidopsis*, rice, etc.)	GASAs	Extracellular	–	Plant growth and development, response to phytohormones, abiotic stresses, defense against pathogen	(Almasia et al. 2008; Conlan et al. 2011; Ko et al. 2007; Muhammad et al. 2019; Pan et al. 2019; Sun et al. 2013; Wang et al. 2009b)

-: Not identified

Functional studies of small peptides in plants have not only broadened our knowledge but have also improved our tools for molecular breeding. However, several issues still need to be considered.

1. Spatiotemporal mining and characterization. Although many types of small peptides have been identified in plants, information on the functions of small peptides discovered to date is largely confined to specific spatial and temporal activities, raising concerns that the repertoire of plant small peptides is limited. One of the possible consequences of these limitations is the lack of rRNA- or circular RNA-encoded peptides reported in plants. Future analysis with wider plant species coverage, plants at more developmental stages and various plant tissues may reveal whether rRNA or circular RNA in plants encodes small peptides.
2. Further exploration of molecular mechanism. The in-depth molecular mechanisms underlying small peptide action are still unclear. For example, although it is known that miPEP binds to the coding sequence of its pri-miRNA of origin, the mechanism by which it activates transcription of the pri-miRNA remains to be determined. In addition, little is known about the mechanisms of lncRNA-derived peptide actions in plants.
3. Conservation analysis. Positive selection leads to accumulation of advantageous genes that are conserved and that possibly control vital processes among different populations. Nevertheless, conservation of small peptides among plant species has not been extensively studied, and the lack of information on conservation has become a barrier to researchers seeking to identify functional small peptides by analyzing high-throughput data.
4. Technical assistance in functional studies. Although recent advancements in proteomics and bioinformatics have enabled quickly identifying small peptides, functional studies are challenging due to the small size of these regulators. Indeed, it is not easy to acquire T-DNA insertional mutants, and any given sgRNA target may not be optimal because of the narrow genome-editing window. Using adenine base editors (ABEs) or cytidine base editors (CBEs) with a wide range of targets may help to overcome this limitation (Tan et al. 2022; Zeng et al. 2020).
5. Production and application of small peptides. Synthetic peptides can be exogenously applied to modulate plant growth and development, which is an environmentally friendly approach compared to use of chemical fertilizers and insecticides. Exogenous small peptides are synthesized mainly by commercial companies, but this has led to high costs. An alternative approach involves utilizing a plant-based expression system to produce small peptides.

We believe that further study of the above-mentioned five aspects will lead to the discovery of more small peptides in plants, and their utilization may additionally benefit biotechnology and agriculture.

## Data Availability

Data sharing is not applicable to this article, as no datasets were generated or analyzed during the current study.
